# Recommendations for the use of biomarkers for head and neck cancer, including salivary gland tumours: a consensus of the Spanish Society of Medical Oncology and the Spanish Society of Pathology

**DOI:** 10.1007/s12094-022-02856-1

**Published:** 2022-06-23

**Authors:** José Trigo, Mónica García-Cosío, Almudena García-Castaño, Montserrat Gomà, Ricard Mesia-Nin, Elena Ruiz-Bravo, Ainara Soria-Rivas, Paola Castillo, Irene Braña-García, Margarita Alberola-Ferranti

**Affiliations:** 1 HC Marbella International Hospital, Spanish Society of Medical Oncology (SEOM), Marbella, Spain; 2 Ramón y Cajal University Hospital, Spanish Society of Pathological Anatomy (SEAP), Madrid, Spain; 3 Marqués de Valdecilla University Hospital, Spanish Society of Medical Oncology (SEOM), Santander, Spain; 4 Bellvitge University Hospital, Spanish Society of Pathological Anatomy (SEAP), Hospitalet de Llobregat, Spain; 5 Catalan Institute of Oncology (ICO), Badalona Applied Research Group in Oncology, Germans Trias i Pujol Research Institute, Spanish Society of Medical Oncology (SEOM), Badalona, Spain; 6 La Paz University Hospital, Spanish Society of Pathological Anatomy (SEAP), Madrid, Spain; 7 Ramón y Cajal University Hospital, Spanish Society of Medical Oncology (SEOM), Madrid, Spain; 8 Clínic de Barcelona Hospital, Spanish Society of Pathological Anatomy (SEAP), Barcelona, Spain; 9 Vall d’Hebron University Hospital, Spanish Society of Medical Oncology (SEOM), Barcelona, Spain; 10 Vall d’Hebron University Hospital, Spanish Society of Pathological Anatomy (SEAP), Barcelona, Spain

**Keywords:** PD-L1, Prognosis, Individualized therapy, Response to treatment, Epstein Barr virus, Human papillomavirus

## Abstract

The treatment of head and neck and salivary gland tumours is complicated and evolves constantly. Prognostic and predictive indicators of response to treatment are enormously valuable for designing individualized therapies, which justifies their research and validation. Some biomarkers, such as p16, Epstein–Barr virus, PD-L1, androgen receptors and HER-2, are already used routinely in clinical practice. These biomarkers, along with other markers that are currently under development, and the massively parallel sequencing of genes, ensure future advances in the treatment of these neoplasms. In this consensus, a group of experts in the diagnosis and treatment of tumours of the head and neck and salivary glands were selected by the Spanish Society of Pathology (Sociedad Española de Anatomía Patológica—SEAP) and the Spanish Society of Medical Oncology (Sociedad Española de Oncología Médica—SEOM) to evaluate the currently available information and propose a series of recommendations to optimize the determination and daily clinical use of biomarkers.

## Introduction

Head and neck squamous cell cancer (HNSCC) is a heterogeneous group of tumours responsible for 5% of cancer cases diagnosed in Spain. It is estimated that each year, approximately 12,000 people develop HNSCC, of whom 1600 die [1]. Treatment modalities include surgery, radiation, chemotherapy, targeted therapies, and immune checkpoint inhibitors (ICIs). For many patients who are cured, the sequelae of treatment can affect their functioning, quality of life, and noncancer-related mortality [2–4]. In this context, indicators of biological behaviour and sensitivity to treatment can be enormously valuable for designing individualized therapies, which justifies the search for predictive biomarkers of response to treatment and prognosis in patients with HNSCC.

Although the human papillomavirus (HPV) prevalence varied from 0 to 85% worldwide, Spain is one of the countries with the lowest proportion of HPV-positive oropharyngeal cancer [5]. Numerous studies have demonstrated the usefulness of biomarkers associated with HPV in oropharyngeal cancer to predict outcomes and select therapies for these patients [6]. In randomized studies, immunotherapy with programmed cell death protein 1 (PD-1) inhibitors such as nivolumab and pembrolizumab has been shown to be effective in patients with advanced HNSCC; however, the percentage of positive tumour cells and the percentage of positive immune cells have been decisive for its indication for approval [7–9]. Promising results have also been published for targeted therapies, such as treatments for patients with fusion of the neurotrophic tyrosine receptor kinase (*NTRK*) gene or mutations in the HRas GTPase gene (*HRAS*) [10, 11]. Table 1 summarizes the main biomarkers currently in use in head and neck cancer.

**Table 1:** Biomarkers in use for different types of head and neck tumours

Biomarker	Tumour type and location	Detection techniques
HPV	Squamous carcinoma in the oropharynx and laterocervical lymph node	IHC for p16 DNA-based tests
EBV	Undifferentiated nonkeratinizing carcinoma in the nasopharynx Squamous carcinoma in the laterocervical lymph node	IHC for LMP-1 ISH for EBERs
PD-L1	Squamous carcinoma (all locations)	IHC (evaluation by CPS)
Androgen receptors	Ductal carcinoma of salivary glands	IHC
HER-2	Salivary gland carcinomas	IHC ISH
NTRK	Salivary gland secretory carcinoma	IHC (Pan-TrK), FISH (break-apart probes) NGS

*HPV* human papillomavirus, *EBV* Epstein–Barr virus, *PD-L1* programmed death ligand 1, *HER-2* human epidermal growth factor receptor 2, *NTRK* neurotrophic tyrosine receptor kinase, *IHC* immunohistochemistry, *ISH* In situ hybridization, *CPS* combined positive score, *LMP-1* latent membrane protein-1, *EBERs* Epstein–Barr virus-encoded small RNAs, *FISH* Fluorescent in situ hybridization, *NGS* next-generation sequencing

This consensus of the Spanish Society of Pathology (Sociedad Española de Anatomía Patológica—SEAP) and the Spanish Society of Medical Oncology (Sociedad Española de Oncología Médica—SEOM) evaluates the available information on prognostic and predictive biomarkers that determine therapeutic choices in different tumours of the head and neck region. The existing information is analysed both for biomarkers currently used in clinical practice and for new biomarkers that are under investigation.

## Squamous carcinoma

### p16 and HPV

Squamous carcinomas of the oropharynx mediated by HPV represent a biologically distinct entity from the classical squamous carcinomas of the oropharynx related to tobacco and alcohol. They have very different epidemiological characteristics and [12], most importantly, they have a much better prognosis, both as localized disease and with regard to recurrence or metastasis [6, 13, 14]. The old TNM/AJCC classifications (TNM Classification of Malignant Tumours/American Joint Committee on Cancer) were not able to highlight prognostic differences by stage, especially for HPV-related tumours between stages I–III. However, the 8th edition of the TNM/AJCC classifies aetiological forms as clinical (cTNM) or pathological (pTNM) stage [15–17], and clearly differentiates the entity of HPV-related oropharyngeal cancer.

In 2018, the College of American Pathologists (CAP) published a consensus recommendation advising screening for high-risk HPV (HR-HPV) for all patients with squamous cell carcinoma of the oropharynx, regardless of morphology [18]. There are several techniques to identify HR-HPV [19]. Immunohistochemistry (IHC) detection of p16 is very sensitive for identifying infections by transcriptionally active HR-HPV. p16 expression is independent of virus subtype and, in the oropharynx, it has a strong correlation with HR-HPV infection. Therefore, the CAP and the American Society of Clinical Oncology (ASCO) recommend its use as an appropriate surrogate marker to identify HR-HPV infection [18, 20]. However, in other locations of the head and neck, p16 shows little correlation with HR-HPV infection [19]; therefore, outside the oropharynx, the use of p16 as a marker is not recommended [18, 20].

In biopsy material, a sample is considered p16 positive when 70–75% of the tumour cells exhibit moderate to strong diffuse and confluent nuclear and cytoplasmic staining [18, 20]. Samples for which 50–75% of cells exhibit staining require a more specific test. If less than 50% of cells exhibit staining, the result is considered negative. In cytology material, staining for p16 can generate false negatives; therefore, molecular analyses would be advised [21].

**Fig. 1:**
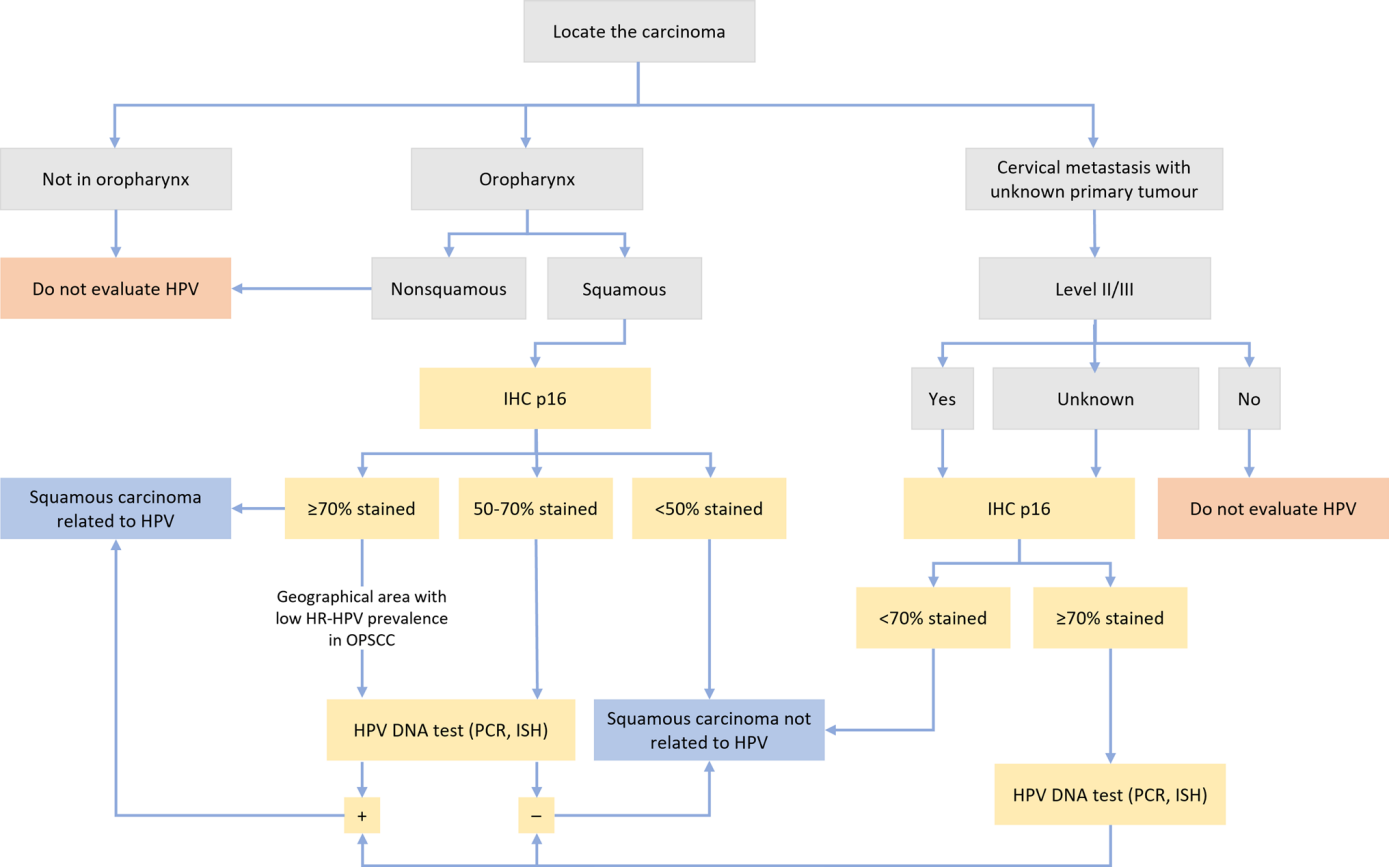
Algorithm of the diagnostic procedure of head and neck tumours according to their location and the presence of HPV. *IHC* immunohistochemistry, *HPV* human papillomavirus, *HR-HPV* high-risk human papillomavirus, *DNA* deoxyribonucleic acid, *PCR* polymerase chain reaction, *ISH* In situ hybridization

Despite the strong correlation of p16 with HR-HPV infection in the oropharynx, staining is very nonspecific and there is a certain percentage of false-positives. The EPIC study confirmed that overall, up to 11% of patients in whom only p16 was assessed had a positive result for p16, but a negative test result for HPV DNA [22]. This percentage increased to 29% in geographic regions where the prevalence of HR-HPV in oropharyngeal squamous cell carcinomas was lower, as in southern European countries. In addition, p16-positive/HPV-negative patients had a prognosis that was intermediate between those for patients with HPV-related and other unrelated tumours. Therefore, and according to what has already been proposed by other working groups [23], a sequential strategy is recommended: first, the use of IHC for the detection of p16 and, if positive, confirmation of the presence of HR-HPV using other molecular tests to avoid undertreatment of false-positives. Figure 1 shows the HPV diagnostic algorithm according to tumour type and location.

The presence of HPV is also an independent predictor of response to any type of treatment [12]. However, the results of studies that evaluated the impact on the efficacy of less aggressive treatments, which aim to reduce acute and chronic toxicity in patients who will foreseeably have longer survival times, are not yet known [24]. From initial published results, treatment with radiotherapy and cetuximab is inferior to standard treatment with radiotherapy and high-dose cisplatin [25, 26].

### PD-L1

Programmed death ligand 1 (PD-L1) is a transmembrane protein that is expressed in haematopoietic and nonhaematopoietic tumour cells. The expression of PD-L1 is one of the mechanisms by which tumour cells escape the antitumour response of the immune system. The interaction of PD-L1 molecules with the PD-1 receptor on T lymphocytes represses the intratumoural lymphocyte response.

The development of oncological therapies that block this pathway of lymphocyte regulation have revolutionized cancer treatment. The results of the Keynote-048 study represent a paradigm shift in the first-line treatment of platinum-sensitive recurrent or metastatic squamous head and neck carcinoma [7]. This study demonstrated that pembrolizumab in monotherapy or in combination with chemotherapy (with platinum and 5-fluorouracil) lengthened overall survival (OS) for patients who expressed PD-L1, with a combined positive score (CPS) ≥ 1, compared to OS for patients who received standard treatment based on cetuximab (EXTREME scheme), especially in patients with a CPS ≥ 20 [27]. However, this benefit was not clear for patients with a CPS < 1, although the combination of pembrolizumab and chemotherapy was considered superior to the EXTREME regimen in this group [28].

Therefore, PD-L1 CPS emerges as a predictive biomarker of response to treatment essential for decision-making in this stage of the disease [29, 30]. The Food and Drug Administration (FDA) has approved pembrolizumab as monotherapy for patients with a CPS ≥ 1 when evaluated with an FDA-approved test and has also approved the combination of pembrolizumab with chemotherapy regardless of the PD-L1 value. In 2020, the European Medicines Agency (EMA) approved the use of pembrolizumab in monotherapy or in combination with chemotherapy as first-line treatment in patients with a CPS ≥ 1 with platinum-refractory disease, without specifying a specific diagnostic test [7–9, 27–32].

In the Checkmate-141 study [9], nivolumab improved survival compared to standard treatment, regardless of PD-L1 positivity as measured by IHC, in platinum-resistant disease. Although the PD-L1 value in routine clinical practice is unknown [29, 30], PD-L1 positivity is a predictive marker of efficacy [32]. In a study with a similar design [8], pembrolizumab only resulted in slight improvement in survival for the entire population, with a greater survival for patients with a tumour proportion score (TPS) ≥ 50. Therefore, the EMA has only approved pembrolizumab for patients with platinum-refractory disease with a TPS ≥ 50.

Currently, there are several staining methods for PD-L1 that use different primary antibodies and different platforms with variable cut-off scores. Some studies suggest that different tests can be interchangeable if the tests are administered with appropriate methodology and cut-off points, but more validation studies are necessary to allow a consensus regarding the determination of PD-L1 expression by IHC [33–37].

The certified diagnostic tests are DAKO (22C3) for pembrolizumab and Ventana (SP263) for durvalumab, with the former being the most widely used. The recommendations for the correct determination of the CPS are provided in Table 2.

**Table 2:** Recommendations and procedure for the calculation of PD-L1 CPS

• The interpretation of the stain should be performed by an experienced pathologist
• IHC staining of PD-L1 can be performed using paraffin samples. To avoid disparate results, it is important to maintain ideal pre-analytical conditions, with a minimum fixation time of 6 h
• Archival samples should be interpreted with caution, as antigenicity may be modified
• Background staining should be < 1 + intensity
• Staining controls should be employed (e.g. tonsil, which should show intense membrane staining in the epithelium of the crypts and weak or moderate membrane staining in macrophages of germinal centres)
• The sample must contain a minimum of 100 tumour cells
• The CPS value is evaluated and is defined by the following formula
$${\text{CPS }} = \frac{{{\text{tumour positive cells, lymphocytes and macrophages}} \times 100}}{{\text{total viable tumour cells}}}$$
• The numerator includes tumour cells of infiltrating squamous cell carcinoma that show convincing staining (visible at 20x) of linear membranes, partial or complete, and lymphocytes or macrophages with staining of any intensity, membrane and/or cytoplasm located near the tumour cells. Inflammatory cells located at a distance from carcinoma or other cell types are not included
• Multinucleated tumour cells that show convincing staining are also included in the numerator
• Staining in areas of squamous cell carcinoma in situ, necrotic areas, or stromal tissue should not be evaluated
• The denominator includes tumour cells from infiltrating HPVs that are positive and negative for PD-L1 staining. Other cell types (e.g., plasma, eosinophils, polymorphonuclear) are not included
• The result is an exact numerical value, with the maximum value being CPS = 100
• The result is reported using three possible categories: CPS < 1 (negative); CPS ≥ 1 (positive) and CPS ≥ 20 (positive)
• In addition to the category, the exact numerical value should be included in the report, as the categories and indications could change in the future

*IHC* immunohistochemistry, *PD-L1* programmed death ligand 1, *CPS* combined positive score

## Nasopharyngeal carcinoma

### Epstein–Barr virus

Nasopharyngeal carcinoma is a relatively infrequent neoplasm that has unique characteristics related to its aetiology, prognosis and treatment compared to those of other head and neck carcinomas [38].

The World Health Organization distinguishes 3 histological subtypes: keratinizing carcinoma, nonkeratinizing carcinoma and basaloid squamous carcinoma. The nonkeratinizing subtype is further subdivided into differentiated and undifferentiated carcinoma. The latter is the most common in East and Southeast Asian countries (> 95%) and is associated in practically all cases with Epstein–Barr virus (EBV) infection [39]. Its prognosis is better than that of keratinizing carcinoma.

This geographical distribution of undifferentiated nonkeratinizing carcinoma is due to the concurrence of several risk factors, such as EBV infection, genetic factors that increase susceptibility to developing carcinoma and environmental factors, such as the consumption of foods high in nitrosamines [38].

Practically all cells of undifferentiated nonkeratinizing carcinomas are infected by EBV. The primary infection by EBV in the epithelial cells of the nasopharynx is lytic, which does not induce cell proliferation or immortalization. For this to occur, evolution to latent EBV infection is facilitated by the presence in premalignant epithelial cells of somatic mutations and alterations in cellular signalling, which activate telomerase and inactivate the tumour suppression genes *RASSF1A* and *p16*. Type II latent EBV infection occurs in undifferentiated nonkeratinizing carcinomas, in which the expression of the *EBNA1*, *LMP1*, *LMP2A*, EBERs and *BART* gene products facilitates the clonal expansion of infected epithelial cells [40]. It is therefore necessary for all nasopharyngeal carcinomas, both for diagnostic and therapeutic purposes, to test for the presence of EBV in the tumour tissue; such tests include IHC for latent membrane protein 1 (LMP-1) and in situ hybridization (ISH) for EBV-encoded small mRNAs (EBERs).

Screening for the presence of EBV in populations who live in areas endemic for undifferentiated nonkeratinizing carcinoma allows the identification of the disease in the initial stages and improves the therapeutic and prognostic outcomes for patients. The serological assessment of the EA-IgA (early antigen), VCA-IgA (capsid antigen) and EBNA1-IgA (nuclear antigen 1) antibodies against EBV has been the most widely used test for this purpose; however, it has been found to have low sensitivity and specificity [41]. More recent studies have demonstrated greater sensitivity in the detection of DNA or RNA in the BamHI-WVEB and EBNA1 genome regions or in EBERs in plasma or serum [42].

## Cervical metastasis of carcinoma of unknown origin

Squamous cell carcinoma of unknown origin of the head and neck is defined as cervical lymph node metastasis without evidence of a primary tumour, after examinations that should include fibroendoscopy and radiological evaluation, preferably positron emission tomography/computerized axial tomography (PET-CAT). These tumours represent between 3 and 10% of head and neck tumours [43, 44], with level II being the most common location, followed by level III.

The initial diagnostic test of choice is fine needle aspiration (FNA), which is a simple, minimally invasive and inexpensive technique that provides a posterior diagnostic biopsy. In addition, it allows immunohistochemical and molecular studies using cell blocks [45]. In cystic metastases, where FNA may not be diagnostic, core needle biopsy guided by ultrasound is recommended [46].

For a metastasis of a carcinoma of unknown origin, an HPV assessment is recommended and, if negative, an EBV assessment should be performed.

### HPV

The presence of HR-HPV should be assessed routinely for levels II and III lymphadenopathies, for which it is common to identify hidden oropharyngeal tumours, especially of the palatine tonsil and base of the tongue. The determination of HPV has prognostic and therapeutic value [46, 47]. Initially, an immunohistochemical assessment of p16 is recommended. p16 is a surrogate marker of the presence of HR-HPV, with high sensitivity and specificity. ASCO recommends confirming positive results with a specific assessment of HR-HPV using DNA-based techniques [20, 46].

### Epstein–Barr virus

Carcinomas of unknown origin associated with EBV are much less frequent. The determination of EBV by the ISH of EBERs is recommended for HPV-negative cases. The presence of EBV usually indicates a nasopharyngeal origin, although it may occasionally be present in tumours that originate in other locations [46].

## Salivary gland tumours

The treatment of choice for salivary gland carcinomas is surgery with adjuvant radiotherapy. However, there are subtypes with rapid progression that complicate surgery and that, until recently, could only be treated with palliative chemotherapy [48]. Currently, androgen receptors, HER-2 and *NTRK* are recommended as possible therapeutic targets.

### Androgen receptors

Salivary ductal carcinoma is a rare tumour that account for 5–10% of all salivary malignancies. It is an aggressive subtype that microscopically resemble high-grade breast duct carcinoma. In salivary glands, 64–98% of ductal carcinomas are positive for androgen receptors [49]. Androgen receptors can be assessed by IHC. Samples are considered positive when ≥ 10% of the nuclei are strongly stained [50]. For ductal carcinomas, positivity is intense and diffuse and correlates with the response to anti-androgenic treatment. There are other subtypes of salivary gland carcinomas that can be positive for androgen receptors, but for these carcinomas, focal and heterogeneous positivity does not correlate with oncogenic development [51].

The AR-V7 androgen receptor variant described in prostate adenocarcinoma causes resistance to anti-androgenic treatment. AR-V7 in ductal carcinoma of the salivary glands also seems indicative of resistance to treatment, although more studies are needed [50]. This variant can be assessed by IHC.

From the therapeutic point of view, complete androgen blockade has shown activity in a single-arm phase II clinical trial evaluating the combination of leuprorelin and bicalutamide in patients with malignant tumours of the salivary gland with androgen receptor positivity, with a response rate of 42% (complete response 11% and partial response 31%) [52]. In this study, there was no restriction due to histological type, although 94% of patients had ductal carcinoma, and only 2 patients (6%) had NOS adenocarcinoma (not otherwise specified) [52]. In a retrospective multicentre study, treatment with adjuvant complete androgen blockade in patients with ductal carcinoma resulted in an improvement in progression-free survival (PFS) [HR 0.138 (95% CI 0.025–0.751, *p* = 0.022)] [53]. Currently, there are no data from randomized studies. The EORTC 1206 (NCT01969578) study is underway. This trial is comparing the activity of complete androgen blockade (bicalutamide and triptorelin) with that of chemotherapy in patients with salivary gland tumours with androgen receptor positivity.

Therefore, in patients with ductal carcinoma or NOS metastatic adenocarcinoma with androgen receptor expression ≥ 10%, treatment should include complete androgen blockade. In addition, for patients with ductal carcinoma and androgen receptor positivity, adjuvant treatment with complete androgen blockade can be considered.

### HER-2

HER-2 is overexpressed in several subtypes of salivary gland carcinomas (ductal carcinoma: 43%; ex pleomorphic adenoma carcinoma: 39%; NOS adenocarcinoma: 13%; and mucoepidermoid carcinoma: 5%) [54]. Its positivity is related to more aggressive tumour behaviour. HER-2 overexpression is measured semiquantitatively by IHC or ISH (fluorescent, chromogenic or silver). Although an attempt has been made to establish a scale to assess IHC results specific for salivary glands, no studies have been validated. Therefore, currently, the same criteria are applied for salivary gland carcinomas as for breast carcinomas [55] (Table 3).

**Table 3:** Criteria for the evaluation of HER-2 immunohistochemistry results for the salivary gland

NEGATIVE (0): No staining or membrane staining is identified in ≤ 10% of tumour cells
NEGATIVE (1 +): weak and partial membrane staining in > 10% of tumour cells
EQUIVOCAL (2 +): moderate/intense complete membrane positivity in > 10% of tumour cells
POSITIVE (3 +): intense complete membrane staining in > 10% of tumor cells
In EQUIVOCAL cases, in situ hybridization should be performed

Several phase II studies support treatment with anti-HER-2 therapy in patients with salivary gland tumours and HER-2 overexpression. One of these nonrandomized single-centre phase II studies evaluated the combination of docetaxel and trastuzumab for patients with metastatic or unresectable ductal carcinoma with HER-2 overexpression, with a response rate of 70% [56]. Two basket trials that evaluated trastuzumab emtansine (T-DM1) and the combination of trastuzumab with pertuzumab have demonstrated the activity of these treatments in patients with salivary gland tumours and HER-2 amplification or overexpression [57, 58]. In a study that evaluated T-DM1 (NCT02675829), 10 patients were treated, of whom only 2 had received anti-HER-2 therapy. The response rate was 90%, and after 12 months of follow-up, the median PFS had not been reached [58]. In the multicentre study MyPathway, a cohort of 18 patients with salivary gland tumours with HER-2 amplification or overexpression was analysed, regardless of histology. The response rate in this group was 63%, the highest of all tumour cohorts classified by location [57].

Therefore, patients with salivary gland tumours with HER-2 overexpression or amplification should be treated with anti-HER-2 therapy, which has response rates like the following regimens: docetaxel-trastuzumab, T-DM1 and trastuzumab-pertuzumab.

### NTRK

The identification of rearrangements in the *NTRK* gene and the development of tyrosine kinase inhibitors have opened a new horizon in the treatment of patients with a neoplasia who present this alteration. *NTRK* plays an important role in the development of secretory carcinomas of the salivary glands (ETV6-NTRK3 fusion in 90–100% of cases). The Canadian guide on tumours with potential *NTRK* fusions recommends testing for androgen receptors and HER-2 in ductal carcinomas and NOS adenocarcinomas and, if negative, testing for *NTRK* (Table 4) [59]. The fusion of this gene can be determined by IHC, fluorescence ISH (FISH) or next-generation sequencing (NGS) [60].

**Table 4:** Advantages and disadvantages of the diagnostic techniques for *NTRK* rearrangement

	IHC	FISH	NGS
Advantages	↑ Sensitivity Accessible Fast	↑ Sensitivity and specificity Accessible	↑ Sensitivity and specificity Concomitant study with other targets
Disadvantages	Unknown specificity Nonstandardized interpretation	3 tests should be used Nonstandardized interpretation	Limited access, expensive ↓ Sensitivity for DNA panels Response time

*IHC* immunohistochemistry, *FISH* fluorescent in situ hybridization, *NGS* next-generation sequencing, *DNA* deoxyribonucleic acid

The European Society for Medical Oncology (ESMO) recommends IHC as a screening tool to determine cases potentially susceptible to fusion. It is advisable to use a Pan-TRK antibody that allows the identification of the three gene products, *NTRK1-3*. The signal can be cytoplasmic, nuclear or mixed, and a result is considered positive if ≥ 1% of cells are stained. The sensitivity of IHC to detect *NTRK* fusions is 96.2% for *NTRK1*, 100% for *NTRK2* and 79.4% for *NTRK3*. In salivary glands, the sensitivity is 88.9%, and the specificity is 52% [61]. It is very important to take care of the pre-analytical sample and include positive controls (such as neural structures which give a positive signal).

For FISH, three probes are necessary for *NTRK1*, *NTRK2* and *NTRK3*, and it is advisable to use *break-apart* probes. Although the interpretation of the results is not standardized, it is recommended to count a minimum of 50 cells with a cut-off point for positive nuclei of 15–20% (separate red and green signals or isolated red signals). The FISH technique allows the confirmation of fusion in cases deemed positive by IHC, nuclear positivity for *NTRK3* by IHC, and the fusion of *NTRK* in a neoplasm whose histology predicts the type of fusion.

Finally, in NGS, it is important to consider that *NTRK* fusions are exclusive and that not all the sequencing panels include the three gene products. In addition, RNA panels have a higher sensitivity than do DNA panels.

There are two drugs directed against *NTRK* fusions authorized by the FDA and the EMA: larotrectinib (selective inhibitor of *NTRK1*, 2 and 3) and entrectinib (inhibitor of *NTRK1*, 2 and 3, *ALK* and *ROS1*). Both drugs have been approved based on basket trials with patients with molecular alterations of interest, regardless of histology. In the aggregate analysis of three phase I/II studies that evaluated larotrectinib, 20 patients with assessable salivary gland tumours were included. In this cohort, the response rate was 90%, with a median duration of response of 35.2 months [62]. In the aggregate analysis of patients with *NTRK* fusions in basket phase I and II studies that evaluated entrectinib, 54 patients evaluable for response were included, of whom 7 had salivary gland tumours (all had secretory carcinomas). No individual data have been reported for patients with salivary gland tumours, but the response rate of the overall population was 57%, with a median duration of response of 10.4 months [63].

Therefore, this committee of experts recommends that for patients with *NTRK* fusions, as determined by NGS, TRK inhibitors (larotrectinib, entrectinib) should be the treatment of choice.

## Other biomarkers in development

### HRAS

The appearance of mutations in *RAS* proto-oncogenes (K, N, H) is involved in oncogenic events; however, the development of targeted therapies against *RAS* has historically been a challenge [64]. *HRAS* mutations affect farnesylation, and tumours with *HRAS* mutations are particularly susceptible to treatment with farnesyl-transferase inhibitors [11]. In HNSCC, *HRAS* is mutated in approximately 4–8% of patients; among these patients, there is a subset of HPV-negative patients without *p53* or caspase-8 mutations and with alterations in DNA copy number (low chromosomal instability) [65]. Tipifarnib is a farnesyl-transferase inhibitor that has been shown to be effective in the KO-TIP-001 phase II trial with patients with incurable solid HNSCC and *HRAS* mutations (m*HRAS*) with a high variant allele frequency (VAF) (m*HRAS* VAF > 20%) [66]. Of the 22 patients evaluated, responses were observed in 55%, with a median PFS of 5.6 months and OS of 15.4 months. The most frequent adverse events related to treatment were anaemia (37%) and lymphopenia (13%). These data support the efficacy of tipifarnib in patients with recurrent or metastatic HNSCC with *HRAS* mutations. *HRAS* mutations can be detected by polymerase chain reaction (PCR, Sanger), but NGS panels (based on RNA or DNA) using tissue, through sequence analysis of the entire coding region, is more suitable because it allows the detection of alterations in multiple genes simultaneously.

### NOTCH1

Sequencing of the HNSCC genome revealed that *NOTCH1* acts as a tumour suppressor gene and that it is the second most frequently mutated gene in this cancer, with an incidence of 15–19% [67]. A structural analysis of *NOTCH1* mutations in HNSCC indicated that the majority are loss-of-function mutations [68]. *NOTCH1* controls the genes involved in early differentiation, having different phenotypic consequences depending on the genetic background of the cancer, including the acquisition of properties like those of progenitor cells. In addition, *NOTCH1* signalling can drive HNSCC tumourigenesis and clinical aggressiveness. The presence of *NOTCH1* mutations detected by NGS can predict the response to treatment with ICIs or phosphatidylinositol-3-kinase (PI3K) inhibitors [69]. The latter are being tested in a clinical trial and, if validated, may lead to the development of the first biomarker associated with targeted therapy in HNSCC.

### PI3K

The PI3K/AKT/mTOR signalling pathway is active in more than 90% of HNSCC cases as a result of *EGFR* activation (47%), mutations in the catalytic alpha subunit of *PIK3CA* (8.6%), *PIK3CA* amplifications (14.2%), PI3K overexpression (27.2%) and *PTEN* mutations (10–15%) [70]. The presence of alterations in this pathway is related to a poor prognosis and resistance to radiotherapy and cytostatic drugs. Preclinical and phase II trials of PI3K/AKT/mTOR inhibitors (buparlisib and alpelisib) have demonstrated efficacy in monotherapy and in combination with chemotherapy [71, 72] and radiotherapy [73–75]. In general, ongoing clinical trials are trying to elucidate the role of AKT and mTOR inhibitors in combination with chemotherapy and radiotherapy and in patients with resistance to them.

### Total mutational load and microsatellite instability

Recently, numerous studies have established the role of neoepitopes as a result of nonsynonymous mutations in tumour cells in the immunological recognition of cancer and in the specific activation of T cells [76]. To determine the total tumour mutational burden (TMB), many studies use NGS based on the complete exome [77]. In HNSCC, TMB has been evaluated in the KEYNOTE-012 trial, with a cut-off of ≥ 102 mutations per exome, and a positive correlation with response to immunotherapy has been observed [78]. Additional data from a cohort of 126 patients who received anti-PD-1/L1 therapy revealed a higher TMB among responders; this was a positive predictor among patients negative for HPV/EBV in the same group. Additionally, microsatellite instability (MSI) was higher among responders [79]. Despite these observations, currently, the subcommittee of the Society of ImmunoTherapy for Cancer (SITC) does not recommend TMB or MSI as biomarkers of response to immunotherapy because they are present in only 1–3% of patients with HNSCC [31].

### Interferon gamma signatures

These signatures were analysed in the KEYNOTE-012 trial, in which the interferon gamma (IFN-γ) signature of 6 genes (gene expression of *IDO1*, *CXCL10*, *CXCL9*, *HLA-DRA*, *STAT1* and *IFN-γ*) was evaluated in biopsies prior to treatment. The results showed that this signature is significantly associated with the response rate and PFS and, due to its high negative predictive value, is a potential biomarker for the exclusion of patients from immunotherapy [78]. The determination of IFN-γ signatures is performed using the RNA of paraffin-embedded tissue, and analysis using a NanoString nCounter system allows the direct multiplex analysis of gene expression [80].

## The role of NGS

In the last decade, massively parallel sequencing of genes by NGS has allowed the sequencing of the cancer genome on an unprecedented scale. NGS has allowed the development of new biomarkers and, more importantly, the identification of patients who are sensitive or resistant to certain therapies [81]. In head and neck carcinomas, the presence of genome alterations has been confirmed, such as mutations in *TP53*, *HER-2*, *CDKN2A*, *PIK3CA*, *PTEN*, *HRAS* and *NOTCH1* [67, 82]. *NOTCH1* is an important oncosuppressive gene and the second most commonly mutated gene in HNSCC [83]. In salivary gland neoplasms, NGS has allowed a new diagnostic characterization, decreasing the number of cases classified as NOS adenocarcinoma and describing actionable alterations in neoplasms with *NTRK* fusions [84, 85]. In addition, through NGS platforms, the state of tumour hypermutation can be determined, which improves the efficacy of immunotherapy for HNSCC.

The genomic alterations collected by the International Cancer Genome Consortium (ICGC), The Cancer Genome Atlas (TCGA), and the Catalogue of Somatic Mutations in Cancer (COSMIC) database provide the most complete source of somatic mutations in cancer to date. Currently, for HNSCC, NGS does not have a clear indication for routine use; however, multigenic sequencing with large panels in clinical research centres that allows the entry of patients into clinical trials is of great interest [86]. There are multiple NGS platforms on the market, all capable of producing precision data, but it is essential to apply guidelines and protocols that guarantee quality control and analytical validity [87]. This would allow, for example, to assess whether the results obtained from fixed and paraffin-embedded tissue samples are consistent with those obtained from frozen samples [88].

In the near future, it is expected that the molecular alterations identified by NGS in HNSCC will result in new therapeutic options, as options have already been identified for some salivary gland neoplasms with clinically treatable mutations, such as *NTRK*.

## Conclusions

The treatment of head and neck and salivary gland tumours is complex and continuously evolving. This consensus describes the most relevant predictive biomarkers and proposes a guide for their determination and interpretation. Some biomarkers, such as p16, EBV, PD-L1, androgen receptors and HER-2, are already applied routinely, and others, such as *NTRK* fusions, have been identified more recently (Table 5). These biomarkers, together with the massively parallel sequencing of genes by NGS, open a hopeful future in the understanding and treatment of these neoplasms. To achieve this objective, the multidisciplinary assessment of these patients and close collaboration between pathologists and oncologists is essential.

**Table 5:** Summary of recommendations

Squamous carcinoma	
Determination of p16 and HPV	Sequential strategy: p16 is first determined by IHC, and if the result is positive, the presence of HR-HPV is confirmed with other molecular tests
Determination of PD-L1	PD-L1 CPS is a predictive biomarker of response to anti-PD-L1 therapies
Nasopharyngeal carcinoma	
Determination of EBV	All nasopharyngeal carcinomas should be assessed for EBV, both for diagnostic and therapeutic purposes, by IHC for LMP-1 or ISH for EBERs
Metastatic squamous carcinoma in the laterocervical lymph node	
Determination of HPV	For levels II and III lymphadenopathies, HR-HPV assessments should be included in routine care. The results have prognostic and therapeutic value
Determination of EBV	The presence of EBV should be determined by ISH for EBERs in HPV-negative cases, which would indicate a nasopharyngeal origin
Salivary gland tumours	
Androgen receptors	In patients with ductal carcinoma or metastatic NOS adenocarcinoma with androgen receptor expression ≥ 10%, treatment should include complete androgen blockade. In patients with ductal carcinoma and positive androgen expression, adjuvant treatment with complete androgen blockade can be considered
HER-2	In patients with salivary gland tumours with HER-2 overexpression or amplification, treatment with anti-HER-2 therapy is recommended, considering the similar response rates between anti-HER-2 therapy and the following regimens: docetaxel-trastuzumab, T-DM1 and trastuzumab-pertuzumab
*NTRK*	In patients with *NTRK* fusions, as determined by NGS, treatment with *NTRK* inhibitors (larotrectinib, entrectinib) is recommended
Other biomarkers in development	
*HRAS*	Potential therapeutic biomarker in a subgroup of HPV-negative patients with low chromosomal instability
*NOTCH1*	Potential predictor of response to treatment with ICIs or PI3K inhibitors
*PI3K*	Potential therapeutic biomarker in a subgroup of patients with resistance to anti-EGFR treatment
TMB and MSI	Potential biomarker of response to immunotherapy, although its routine evaluation is not currently recommended
Gamma interferon signatures	Potential biomarker for the exclusion of patients from immunotherapy due to its high negative predictive value
Next-generation sequencing	NGS has allowed the development of new biomarkers and the identification of patients sensitive or resistant to certain therapies

*HPV* human papillomavirus, *HR-HPV* high-risk human papillomavirus, *EBV* Epstein–Barr virus, *IHC* immunohistochemistry, *ISH* In situ hybridization, *CPS* combined positive score, *PD-L1* programmed death ligand 1, *PI3K* phosphatidylinositol-3-kinase, *LMP-1* latent membrane protein-1, *EBER* Epstein–Barr virus-encoded small RNAs, *HER-2* human epidermal growth factor receptor 2, *NOTCH1* notch homologue 1, *NTRK* neurotrophic tyrosine receptor kinase, *NGS* next-generation sequencing, *ICIs* immune checkpoint inhibitors

## Data Availability

Not applicable.
