# Correction to: Recommendations for the use of biomarkers for head and neck cancer, including salivary gland tumours: a consensus of the Spanish Society of Medical Oncology and the Spanish Society of Pathology

**DOI:** 10.1007/s12094-022-02954-0

**Published:** 2022-09-24

**Authors:** José Trigo, Mónica García-Cosío, Almudena García-Castaño, Montserrat Gomà, Ricard Mesia-Nin, Elena Ruiz-Bravo, Ainara Soria-Rivas, Paola Castillo, Irene Braña-García, Margarita Alberola-Ferranti

**Affiliations:** 1 HC Marbella International Hospital, Spanish Society of Medical Oncology (SEOM), Marbella, Spain; 2 Ramón y Cajal University Hospital, Spanish Society of Pathological Anatomy (SEAP), Madrid, Spain; 3 Marqués de Valdecilla University Hospital, Spanish Society of Medical Oncology (SEOM), Santander, Spain; 4 Bellvitge University Hospital, Spanish Society of Pathological Anatomy (SEAP), Hospitalet de Llobregat, Spain; 5 Catalan Institute of Oncology (ICO), Badalona Applied Research Group in Oncology, Germans Trias i Pujol Research Institute, Spanish Society of Medical Oncology (SEOM), Badalona, Spain; 6 La Paz University Hospital, Spanish Society of Pathological Anatomy (SEAP), Madrid, Spain; 7 Ramón y Cajal University Hospital, Spanish Society of Medical Oncology (SEOM), Madrid, Spain; 8 Clínic de Barcelona Hospital, Spanish Society of Pathological Anatomy (SEAP), Barcelona, Spain; 9 Vall d’Hebron University Hospital, Spanish Society of Medical Oncology (SEOM), Barcelona, Spain; 10 Vall d’Hebron University Hospital, Spanish Society of Pathological Anatomy (SEAP), Barcelona, Spain

## Correction to: Clinical and Translational Oncology (2022) 24:1890–1902 10.1007/s12094-022-02856-1

In this article the statement in the Funding information section was incorrectly given as ‘SEOM and SEAP acknowledge the financial support for this project in the form of unrestricted collaboration in the logistics from AstraZeneca’ and should have read ‘SEOM and SEAP acknowledge the financial support for this project in the form of unrestricted collaboration in the logistics from Merck’.

